# Characteristics, Outcomes, and Cost Patterns of High-Cost Patients in the Intensive Care Unit

**DOI:** 10.1155/2018/5452683

**Published:** 2018-09-02

**Authors:** Peter M. Reardon, Shannon M. Fernando, Sasha Van Katwyk, Kednapa Thavorn, Daniel Kobewka, Peter Tanuseputro, Erin Rosenberg, Cynthia Wan, Brandi Vanderspank-Wright, Dalibor Kubelik, Rose Anne Devlin, Christopher Klinger, Kwadwo Kyeremanteng

**Affiliations:** ^1^Division of Critical Care Medicine, University of Ottawa, Ottawa, ON, Canada; ^2^Department of Emergency Medicine, University of Ottawa, Ottawa, ON, Canada; ^3^Ottawa Hospital Research Institute, Ottawa, ON, Canada; ^4^School of Epidemiology, Public Health and Preventive Medicine, University of Ottawa, Ottawa, ON, Canada; ^5^Institute for Clinical and Evaluative Sciences, University of Ottawa, Ottawa, ON, Canada; ^6^Department of Medicine, University of Ottawa, Ottawa, ON, Canada; ^7^Division of Palliative Care Medicine, University of Ottawa, Ottawa, ON, Canada; ^8^School of Psychology, University of Ottawa, Ottawa, ON, Canada; ^9^School of Nursing, University of Ottawa, Ottawa, ON, Canada; ^10^Division of Vascular Surgery, University of Ottawa, Ottawa, ON, Canada; ^11^Department of Economics, University of Ottawa, Ottawa, ON, Canada; ^12^Factor-Inwentash, Faculty of Social Work, University of Toronto, Toronto, ON, Canada

## Abstract

**Background:**

ICU care is costly, and there is a large variation in cost among patients.

**Methods:**

This is an observational study conducted at two ICUs in an academic centre. We compared the demographics, clinical data, and outcomes of the highest decile of patients by total costs, to the rest of the population.

**Results:**

A total of 7,849 patients were included. The high-cost group had a longer median ICU length of stay (26 versus 4 days, *P* < 0.001) and amounted to 49% of total costs. In-hospital mortality was lower in the high-cost group (21.1% versus 28.4%, *P* < 0.001). Fewer high-cost patients were discharged home (23.9% versus 45.2%, *P* < 0.001), and a large proportion were transferred to long-term care (35.1% versus 12.1%, *P* < 0.001). Patients with younger age or a diagnosis of subarachnoid hemorrhage, acute respiratory failure, or complications of procedures were more likely to be high cost.

**Conclusions:**

High-cost users utilized half of the total costs. While cost is associated with LOS, other drivers include younger age or admission for respiratory failure, subarachnoid hemorrhage, or after a procedural complication. Cost-reduction interventions should incorporate strategies to optimize critical care use among these patients.

## 1. Background

Care provided in the Intensive Care Unit (ICU) is expensive. On average, the daily cost of an ICU bed is threefold higher than a bed in a general ward [1]. Overall, critical care accounts for a significant portion of health care costs, as 11% of hospital admissions now incorporate a stay in the ICU [1]. Additionally, ICU costs are expected to rise due to an aging population and the increasing severity of illness among hospitalized patients [2, 3]. Recent data reveal a 12% increase in ICU utilization over a 6-year period, with up to one-third of these patients requiring invasive mechanical ventilation [1]. This escalating demand for critical care places financial strain on health care systems, prompting the need for strategies to mitigate costs.

It has been well described in the literature that a small proportion of patients account for a disproportionate amount of health care spending [4–10]. Identifying and describing these patients may present an opportunity for focused interventions to reduce spending. However, there is a paucity of literature describing the characteristics of these patients in the general ICU setting [11, 12]. Welton et al. retrospectively examined a cohort of adult ICU patients admitted to a single academic centre in the United States [11]. Although they separated out the top decile of high-resource users and described total costs, their study did not categorize costs or outline the specific use of resources. They did not perform any regression analysis to determine what patient factors are associated with high cost.

This study aims to further describe high-cost users in the ICU by identifying patient factors, cost patterns, and outcomes associated with high cost. A better understanding of the high-resource user may help care providers anticipate resource demands and provide an opportunity to implement interventions to reduce costs without compromising the quality of care.

## 2. Methods

This is a retrospective observational cohort study conducted with the approval of the Ottawa Health Science Network Research Ethics Board.

### 2.1. Study Population

The Ottawa Hospital (TOH) is a tertiary care, academic health sciences hospital, which serves as a referral centre for approximately 1.3 million people in Eastern Ontario, Canada. There are two tertiary care ICUs with 28 beds at each site. The ICU at TOH is a closed model, requiring consultation with an intensivist prior to admission. Discharge planning is conducted as a team approach, requiring approval from the attending intensivist, as well as acceptance from a consulting service who assumes responsibility once the patient is discharged. Upon discharge, the ICU team conducts routine follow-up until the patient is deemed stable by the intensivist.

The study population included all adult patients with at least one admission to an ICU at The Ottawa Hospital between January 1, 2011, and December 31, 2014. For patients with more than one hospital admission with an ICU stay during the study period, only the outcomes and cost for the first admission were considered. The patients were followed up for the length of their hospitalization until discharge or up to one year (whichever comes first), which included any transfers out of or back into the ICU. The one-year limit was added to reduce the weighted effect of prolonged individual admissions on study data.

### 2.2. Data Sources

All data for the study were obtained from the Data Warehouse at The Ottawa Hospital. The Data Warehouse contains information from several information systems including the patient registration system, clinical data repository, case-costing system, and patient abstracts for multiple encounter types [13]. Case costing at The Ottawa Hospital is determined using the system developed by the Ontario Case Costing Initiative [14], based on the Canadian Institute for Health Information Management Information Systems Guidelines [15]. The case-costing system provides detailed information regarding the direct and indirect costs incurred by each patient during their admission.

Direct costs are all expenses to the hospital with fee codes linked to the patient chart, including salaries and benefits for the unit producing and management staff, equipment, and screening and procedure materials. The Data Warehouse uses a unit value of cost per minute of service for all staff, materials, and equipment within the hospital and calculates the direct cost according to the time it was utilized for the patient. Indirect costs are any overhead operational fees associated with the service being provided to the patient such as the cost of the room they occupy per hour. External care facilities costs to which patients may be discharged were not included in this analysis. All costs are reported in Canadian dollars (CDN).

### 2.3. Variables

From the Data Warehouse, we used the patient registry file and hospital abstracts to determine the demographic and clinical characteristics of included patients. We obtained detailed admission data from the hospital discharge abstracts including admission and discharge dates, length of stay (LOS), level of care provided (ward versus ICU), most responsible diagnosis (MRDx), and disposition. Hospital discharge abstracts were also used to identify previous ICU and Emergency Department (ED) encounters within the 12 months before the index admission. Comorbid conditions were summarized and presented as the Elixhauser comorbidity index [16, 17], which uses coded information based on the International Classification of Diseases, Version 10 (ICD-10-CA). These data are collected and coded routinely for every admission by the health records department. Patients with missing data were excluded from the analysis.

Patients were characterized as either “high cost” or “non-high cost.” The high-cost group was defined as the top 10% of patients incurring the largest total (direct and indirect) admission costs. The primary objective of this study was to compare the high-cost users to the remaining 90% of patients according to patient characteristics, primary diagnoses, and comorbidity score.

### 2.4. Statistical Analysis

Patient demographics such as age, sex, and Elixhauser comorbidity score, as well as the disposition (e.g., home, acute care transfer, and long-term care transfer) and MRDx, in the high-cost and non-high-cost groups were compared using the chi-square test or Fisher's exact test, with the level of significance of 0.05. Continuous variables were assessed with the Mann–Whitney *U* test. Age was categorized into four categories: aged 18 to 45, 46 to 69, 70 to 79, and 80 or older. Univariate analyses were conducted to compare patient characteristics between the high-cost cohort and the remaining 90% of patients. Cost was calculated by total costs, indirect and direct costs, as well as a cost breakdown to view the components responsible for the cost of the high-cost and non-high-cost groups.

We also performed a multivariate logistic regression to determine factors associated with the high-cost status and estimated an adjusted odds ratio (OR). The inclusion of variables in the regression analysis was guided by previous studies and subject to availability of the variables in our database. Our regression model included age category, sex, Elixhauser comorbidity score, and MRDx. We performed a subgroup analysis of patients who died during hospitalization using the same logistic regression approach.

## 3. Results

There were 7,849 patients admitted to the ICU during the four-year study period with 786 in the high-cost group and 7,063 in the non-high-cost group. Direct costs, representing approximately 75% of total costs for the study period, had a median value of $148,328 (IQR $114,008–$224,611) in the high-cost group, compared to $20,407 (IQR $10,977–$37,750) in the non-high-cost group. The characteristics of the included study patients are presented in Table 1. The mean age was 60.7 years (SD 16.3) in the high-cost group and 62.3 years (SD 17.3) in the non-high-cost group (*P*=0.012). The age categories 18–45, 46–69, and 70–79 were similar between groups; however, there were fewer patients aged greater than 80 years in the high-cost group (10.8% versus 16.4%, *P*=0.007). There were high rates of congestive heart failure (14.8 versus 9.1, *P* < 0.001), chronic obstructive pulmonary disease (19.0 versus 12.4, *P* < 0.001), and baseline renal failure (8.9 versus 5.3, *P* < 0.001) in the high-cost group. Sex and the number of Emergency Department visits within the preceding 12 months were similar between groups. There were statistically more ICU encounters in the preceding 12 months in the high-cost group (1.3 versus 1.0, *P* < 0.001). For the high-cost group, the median total LOS was 68 days (IQR 49–103) versus 10 days (IQR 4–20) for the non-high-cost group. A median of 26 days (IQR 17–38) were spent in the ICU in the high-cost group compared to 4 (IQR 2–8) ICU days for those in the non-high-cost group (*P* < 0.001).

In terms of disposition, only 23.9% of patients in the high-cost cohort were ultimately discharged directly home from hospital, compared to 45.2% in the non-high-cost group (*P* < 0.001). A large proportion of high-cost patients, 35.1%, were transferred from hospital to long-term care, relative to 12.1% in the non-high-cost group (*P* < 0.001). In-hospital mortality was significantly lower in the high-cost cohort at 21.1%, compared to 28.4% of the non-high-cost group (*P* < 0.001).

Hospital costs are shown in Table 2. The high-cost group was responsible for 49% percent of total costs. Direct costs had a median value of $148,328 (IQR $114,008–$224,611) in the high-cost group, compared to $20,407 (IQR $10,977–$37,750) in the non-high-cost group. The mean cost per day was also significantly increased in the high-cost group $3645 (SD 1915), relative to the non-high-cost group $3327 (SD 2159) (*P* < 0.001). Cost breakdown shows the highest proportion of cost is generated from the ICU and nursing care for both groups.

The results of the multivariate logistic regression (Table 3) show that compared to patients aged 18–45, all older age categories are less likely to be in the high-cost group. When compared to patients sent home, patients are more likely to be high-resource users if they were transferred out of ICU to an inpatient acute care setting (adjusted OR 2.49, 95% CI 2.02–3.08). Conversely, patients who died were less likely to be in the high-resource group (adjusted OR 0.65, 95% CI 0.52–0.81). Patients transferred to continuing care were of similar likelihood between cost groups. With regard to MRDx, patients admitted with ischemic stroke (adjusted OR 0.53, 95% CI 0.29–0.99), heart failure (adjusted OR 0.39, 95% CI 0.29–0.99), or AAA and dissection (adjusted OR 0.60, 95% CI 0.41–0.87), or with an overdose (adjusted OR 0.08, 95% CI 0.02–0.34) were significantly less likely to be within the high-resource group. Patients with an MRDx of acute respiratory failure (adjusted OR 2.44, 95% CI 1.88–3.18), subarachnoid hemorrhage (adjusted OR 2.18, 95% CI 1.47–3.24), complications of procedures (adjusted OR 1.80, 95% CI 1.33–2.44), malignancy (adjusted OR 1.56, 95% CI 1.23–1.98), or sepsis (adjusted OR 1.55, 95% CI 1.26–1.91) were more likely to be in the high-cost group.

Among decedents (Table 4), when compared to patients aged 18–45 years, patients aged greater than 80 years were less likely to be in the high-cost group (adjusted OR 0.37, 95% CI 0.28–0.50). Sex and Elixhauser comorbidity score were not associated with the high-cost status in the regression model. Patients with an MRDx of traumatic intracranial injury were less likely to be in the high-cost group (adjusted OR 0.25, 95% CI 0.08–0.82), while patients with complications of procedures (OR 2.85, 95% CI 1.52–5.33), acute respiratory failure (OR 2.25, 95% CI 1.37–3.68), or malignancy (OR 1.65, 95% CI 0.08–2.67) were more likely to be in the high-cost group. There were no deaths among high-cost patients with an MRDx of acute ischemic stroke or an overdose.

## 4. Discussion

This is a retrospective study conducted at a large academic centre to examine the demographics, characteristics, and outcomes of high-cost users in the ICU. In our analysis, the top 10% of high-cost users amounted to 49% of total costs. ICU LOS and hospital LOS were significantly longer in the high-cost group. Younger age was significantly associated with high cost. Although high-cost users were less likely to be discharged home, they were also less likely to die, with the highest proportion being discharged to long-term care, and an MRDx of subarachnoid hemorrhage (SAH), acute respiratory failure (ARF), and complications of procedures had the strongest association with high cost, while an MRDx of ischemic stroke, heart failure, and overdose was the least costly.

Similar to the previous literature [18–20], LOS appears to be a major driver of high cost in this study as reflected by the substantially longer acute length of stay in the high-cost group. Currently in Ontario, the average daily cost in the ICU at a teaching hospital is $4,186, while an acute care bed is valued at $1,492 per day [1]. The magnitude of cost difference between levels of care highlights the critical importance of patient flow within the hospital and also appropriate use of specialty care units to maximize the utility of hospital resources and reduce overall costs [21, 22]. Providing critical care outside of the ICU has been proposed as a solution to facilitate early discharge and avoid unnecessary admissions. However, recent discussions have had mixed reviews, and definitive evidence is lacking [21, 23].

In addition, cost per day was significantly higher for the high-cost group. As an MRDx of ARF and SAH was most strongly associated with high cost, it is likely that daily cost was influenced by the type of interventions and support required for their treatment. It has been well described in the literature that mechanical ventilation is of noteworthy contribution to ICU costs [20, 24], leading to higher daily cost for ARF patients, while the care of SAH requires complex neuroimaging and procedural therapy which drive higher costs [25].

In this study, complications of procedures demonstrated a strong association with high cost. The association was stronger when only decedents were assessed. The high cost associated with procedural complications, particularly surgical complications, has been well documented in the literature with often substantial increases in costs [26–28]. Causes of this are unclear, but possibly a sense of responsibility from the physician can lead to persistent patient care at times when it is no longer indicated. Further study is warranted in order to explore how the development of targeted investigations, such as earlier palliative care consultation [29–31], may benefit these patients and reduce ICU costs.

Overall, mortality was lower for the high-cost group. This is in contrast to previous studies demonstrating increased costs among those dying in the ICU [24]. As cost appears to be heavily influenced by LOS, those with severe pathologies may have died sooner, reducing their impact on costs. Conversely, our high-cost group was younger. Patients aged greater than 80 years were the least likely to be associated with high cost. This may have impacted our mortality rates as advanced age has been linked to higher mortality rates [32, 33]. Interestingly, this association of younger age and high cost has not been previously demonstrated. Other studies have shown either no association or a trend towards increasing costs for older patients [12, 34]. Although not significant, the other (non-ICH) trauma groups trended towards higher costs. A younger cohort of patients with traumatic injuries requiring surgical intervention and prolonged recovery may explain these findings.

## 5. Limitations

This is a database study with limited patient-level information. Future studies with more detailed data on medical comorbidities and their severity and interventions provided during hospitalization would help identify targets for cost-reduction strategies. Unfortunately, illness severity scores (e.g., APACHE and SAPS) were not available in our database and could not be included for comparison between groups. It should also be noted that regression analysis of diagnoses associated with high cost was conducted with MRDx, as admitting diagnosis was not available for study. In some cases, the admitting diagnosis may have differed from the postdischarge MRDx.

With respect to cost information, this was a hospital database and thus physician billing is not included. We also did not have data-itemizing procedures such as dialysis and tracheostomy, and although we have the total costs, daily costs were not available for presentation. Furthermore, if patients had multiple admissions during the study period, this analysis only took into account the first admission for consistency. Multiple admissions during the study period may have increased overall costs for an individual patient and moved them into an alternate cost group. This is also a single-centre study, so generalizability to different practice settings may be limited. Additionally, the retrospective study design limits our findings to associations and does not imply causation. Finally, functional data on patients before or after admission were not available, which could affect treatment plans and outcomes.

## 6. Conclusion

In a population of high-cost ICU patients, we found that the top 10% of patients accounted for half of the cost. Patients in the high-cost group were less likely to be discharged home and more likely to be discharged to long-term care. While cost is associated with LOS, other drivers include younger age or admission for respiratory failure, subarachnoid hemorrhage, or after a procedural complication. Future cost-reduction interventions should incorporate strategies to optimize critical care use among these patients and facilitate efficient transfer out of the ICU when clinically stable.

## Figures and Tables

**Table 1:** Patient characteristics.

	High cost (*n*=786)	Non-high cost (*n*=7063)	*P* value
Age, mean years (SD)	60.7 (16.3)	62.3 (17.3)	0.012
Category (%)			
18–45	17.3	15.9	0.274
46–69	51.2	46.3	0.113
70–79	20.7	21.4	0.845
80+	10.8	16.4	0.007
Sex, male	59.6	56.1	0.063
Elixhauser comorbidity score (total)	6.12	5.28	<0.001
Congestive heart failure	14.8	9.1	<0.001
Pulmonary circulation disorder	5.6	3.5	0.003
Peripheral vascular disorder	10.1	10.8	0.527
Chronic obstructive pulmonary disease	19.0	12.4	<0.001
Diabetes	25.7	26.4	0.658
Renal failure	8.9	5.3	<0.001
Liver disease	6.6	5.4	0.185
Metastatic cancer	5.5	7.2	0.075
Solid tumour without metastases	13.3	13.3	0.988
Emergency Department visits within 12 months, mean	3.1	2.8	0.201
Intensive Care Unit admissions within 12 months, mean	1.3	1.0	<0.001
LOS, median days (IQR)			
Acute LOS	38 (17–74)	4 (1–11)	<0.001
ICU LOS	26 (17–38)	4 (2–8)	<0.001
Total LOS	68 (49–103)	10 (4–20)	<0.001
Disposition (%)			
Home	23.9	45.2	<0.001
Signed out against medical advice	0.1	0.7	0.029
Transferred to acute care	21.1	13.9	0.003
Transferred to long-term care	35.1	12.1	<0.001
In-hospital mortality	21.1	28.4	<0.001

SD: standard deviation; LOS: length of stay; IQR: interquartile range.

**Table 2:** Cost comparison by high-cost status (Canadian dollars (CDN)).

Cost breakdown	High cost (*N*=786)	Non-high cost (*N*=7063)	*P* value
Total cost per patient, median CDN (IQR)	196,766	27,120	
Direct cost per patient, median CDN (IQR)	148,328 (114,008–224,611)	20,407 (10,977–37,750)	
Cost per day, median CDN (IQR)	3,213 (2,313–4,549)	2,883 (2,079–3,919)	<0.001
Cost per day, mean CDN (SD)	3,645 (1,915)	3,327 (2,159)	<0.001
Direct cost allocation (%)			
Endoscopy	0.2	0.2	
Food services	2.2	2.1	
Health professional services	3.4	2.4	
Imaging	2.8	3.8	
Laboratory	4.0	5.6	
Nursing	36.4	24.8	
Operating room	2.0	5.4	
Pharmacy	7.5	5.4	
Postanaesthetic care unit	0.2	0.5	
Intensive Care Unit	41.3	49.6	

SD: standard deviation; IQR: interquartile range.

**Table 3:** Multivariate logistic regression for variables associated with high cost among all patients.

Characteristics	OR	95% confidence intervals	*P* value
Age			
18–45	Reference		
46–69	0.75	0.61–0.92	0.006
70–79	0.66	0.52–0.84	0.001
80+	0.37	0.28–0.50	<0.001

Sex			
Male	1.23	0.00–1.42	0.016

Discharge disposition			
Home	Reference		
Transfer acute care	2.49	2.02–3.08	<0.001
Transfer continuing care	1.59	0.70–3.61	0.263
In-hospital mortality	0.65	0.52–0.81	<0.001

Elixhauser comorbidity score			
Elixhauser comorbidity score (total)	1.02	1.00–1.03	0.006

Most responsible diagnosis			
Sepsis	1.55	1.26–1.91	<0.001
Malignancy	1.56	1.23–1.98	<0.001
Subarachnoid hemorrhage	2.18	1.47–3.24	<0.001
Other nontraumatic ICH	0.95	0.55–1.64	0.856
Ischemic stroke	0.53	0.29–0.99	0.046
Heart failure	0.39	0.16–0.96	0.040
AAA and dissection	0.60	0.41–0.87	0.007
Pneumonia	0.98	0.59–1.64	0.940
COPD	0.85	0.54–1.35	0.498
Acute respiratory failure	2.44	1.88–3.18	<0.001
Vascular disorders of the intestine	0.82	0.41–1.63	0.567
Liver	0.62	0.32–1.20	0.158
Renal failure	0.49	0.22–1.14	0.097
Traumatic intracranial injury	1.03	0.73–1.46	0.863
Other traumas	1.34	0.99–1.82	0.029
Overdoses	0.08	0.02–0.34	<0.001
Complications of procedures	1.80	1.33–2.44	<0.001

Most responsible diagnosis groups are compared to a reference group made up of all other diagnoses.

**Table 4:** Multivariate logistic regression for variables associated with high cost among decedents.

Characteristics	OR	95% confidence intervals	*P* value
Age			
18–45	Reference		
46–69	0.63	0.38–1.02	0.061
70–79	0.63	0.37–1.05	0.075
80+	0.24	0.13–0.44	<0.001

Sex			
Male	1.39	1.03–1.87	0.032

Elixhauser comorbidity score			
Elixhauser comorbidity score (total)	1.00	0.98–1.02	0.867

Most responsible diagnosis			
Sepsis	1.21	0.80–1.80	0.365
Malignancy	1.68	1.05–2.67	0.030
Subarachnoid hemorrhage	0.35	0.08–1.48	0.152
Other nontraumatic ICH	—		
Ischemic stroke	—		
Heart failure	—		
AAA and dissection	2.20	0.87–5.53	0.095
Pneumonia	0.90	0.35–2.33	0.828
COPD	1.00	0.41–2.40	0.995
Acute respiratory failure	2.25	1.37–3.68	0.001
Vascular disorders of the intestine	0.44	0.10–1.85	0.260
Liver	0.51	0.15–1.70	0.273
Renal failure	0.62	0.08–4.75	0.643
Traumatic intracranial injury	0.25	0.08–0.82	0.022
Overdoses	—		
Complications of procedures	2.85	1.52–5.33	0.001

Most responsible diagnosis groups are compared to a reference group made up of all other diagnoses.

## Data Availability

The data used to support the findings of this study are available from the corresponding author upon request.
